# Sequential Fixation Behavior in Road Marking Recognition: Implications for Design

**DOI:** 10.3390/jemr18050059

**Published:** 2025-10-21

**Authors:** Takaya Maeyama, Hiroki Okada, Daisuke Sawamura

**Affiliations:** 1 Graduate School of Health Sciences, Hokkaido University, Kita 12-jo Nishi 5-chome, Kitaku, Sapporo 060-0812, Japan; maeyama.takaya.n9@elms.hokudai.ac.jp; 2 Department of Rehabilitation Sciences, Hokkaido University, Sapporo, Kita 12-jo Nishi 5-chome, Kitaku, Sapporo 060-0812, Japan; d.sawamura@pop.med.hokudai.ac.jp

**Keywords:** driving, road marking, fixation, driving speed, visual recognition, on-board movies

## Abstract

This study examined how drivers’ eye fixations change before, during, and after recognizing road markings, and how these changes relate to driving speed, visual complexity, cognitive functions, and demographics. 20 licensed drivers viewed on-board movies showing digit or character road markings while their eye movements were tracked. Fixation positions and dispersions were analyzed. Results showed that, regardless of marking type, fixations were horizontally dispersed before and after recognition but became vertically concentrated during recognition, with fixation points shifting higher (*p* < 0.001) and horizontal dispersion decreasing (*p* = 0.01). During the recognition period, fixations moved upward and narrowed horizontally toward the final third (*p* = 0.034), suggesting increased focus. Longer fixations were linked to slower speeds for digits (*p* = 0.029) and more characters for character markings (*p* < 0.001). No significant correlations were found with cognitive functions or demographics. These findings suggest that drivers first scan broadly, then concentrate on markings as they approach. For optimal recognition, simple or essential information should be placed centrally or lower, while detailed content should appear higher to align with natural gaze patterns. In high-speed environments, markings should prioritize clarity and brevity in central positions to ensure safe and rapid recognition.

## 1. Introduction

Driving is a critical activity that supports daily living, employment [1], personal independence [2], and overall quality of life [3], making it socially essential. Simultaneously, driving is a complex task that demands continuous visual attention to detect and avoid potential hazards such as surrounding vehicles, road obstacles, and pedestrians [4], as well as to recognize traffic and guide signs accurately [5,6]. Inadequate visual recognition can significantly increase the risk of traffic accidents [7]. Therefore, accurate visual recognition of traffic signs, road markings, and other environmental cues is vital for safe driving.

Among these cues, road markings—such as digit-based indicators of speed limits and character-based warnings of road conditions—play an essential role [8,9,10]. Because road markings occupy a substantial portion of the driver’s field of view, they serve as an effective medium for conveying important information [11]. They contribute to safer driving by encouraging speed reduction and hazard avoidance, particularly at intersections and on curves [12,13,14]. Consequently, road markings must be designed for quick and accurate recognition.

However, fundamental data on the fixation behavior of drivers when recognizing digit- and character-based road markings remain lacking, and whether current road markings are consistently easy to recognize is unclear. Previous studies have shown that both the amount of information on signage and driving speed affect the recognition of roadside or overhead guide signs [5,6,15], suggesting that similar factors may influence the road marking recognition. Therefore, understanding fixation behavior during the recognition of these markings is essential to improving their design.

In this study, we used on-board video recordings and eye-tracking technology to examine how drivers recognize digit and character road markings. In this study, character road markings are defined as those consisting of hiragana, katakana, or kanji. We analyzed the differences in fixation behavior between these two types and investigated how fixation is influenced by driving speed and the number of stimuli.

Analysis 1 examined differences in the fixation position and dispersion before, during, and after the visual recognition of road markings.

Analysis 2 divided the recognition period into three phases (first, middle, and final thirds) to assess changes in fixation behavior over time.

Analysis 3 explored the correlations between the fixation duration and count with the driving speed and number of visual stimuli based on previous studies [5,15].

Analysis 4 investigated the associations among fixation behavior, cognitive functions, and basic demographics, referencing previous research on guide sign recognition [6,16].

Through these analyses, this study aimed to clarify how current road markings are visually processed and to offer insights for the development of safer, more effective road marking designs. Two types of road marking were used as stimuli: digit-based markings indicating speed limits and character-based markings conveying warning messages to drivers. This study is positioned as preliminary work aimed at exploring fixation behaviors during the viewing of on-board movies, prior to conducting experiments using driving simulators or real vehicles. It represents an initial attempt to clarify drivers’ visual search behavior in actual driving scenes within the framework of exploratory research. Examining the total amount of visual attention in such contexts is meaningful, as it can indicate cognitive effort and information-processing load, even in the absence of active driving operations [17,18,19].

## 2. Materials and Methods

### 2.1. Participants

This study is part of a broader research project investigating the relationship between cognitive functions and eye movements during the recognition of road markings while driving, with a particular focus on individuals with impaired driving abilities, including those with mental disabilities. In this paper, we report the exploratory and preliminary analysis that was conducted with a healthy control group. Some data analyzed in this study overlap with those from our previous research [6].

The required sample size was calculated using G*Power version 3.1.9.7 [20] for a repeated-measures ANOVA (within-subject factors), assuming an effect size of f = 0.3, power of 0.8, alpha of 0.05, two groups, and three measurements. This resulted in a target sample size of 20 participants.

The participants were selected from a previous study based on the following inclusion criteria: (1) aged 20 to 59 years and holding a valid driver’s license; (2) normal or corrected-to-normal visual acuity of 0.6 or higher (equivalent to logMAR 0.22 or lower); (3) no history of neurological or psychiatric disorders; (4) no history of epilepsy; and (5) no ophthalmologic conditions, including cataracts or glaucoma. Finally, 20 participants (eight males and 12 females) were analyzed. Although the sample size is relatively small, we consider it adequate for this exploratory study aimed at providing initial insights into the relationship between road marking recognition and fixation behavior. The mean age was 36.7 ± 13.8 years (range: 21–59), the mean driving history was 16.6 ± 14.5 years (range: 0–40), and the mean educational history was 15.4 ± 1.8 years (range: 12–20). In this study, “driving history” included both independent driving after license acquisition and on-road training during the licensing process. All participants only used cars for daily living purposes and had no professional driving experience.

### 2.2. Apparatus

The visual stimuli were presented on a 24-inch monitor (ProLite B2480HS, iiyama, Nagano, Japan; resolution: 1920 × 1080, brightness: 300 cd/m^2^), and eye movements were recorded using an eye tracker (Tobii X60, Tobii AB, Danderyd, Sweden) at a sampling rate of 60 Hz. The eye-tracking data were analyzed using Tobii Studio version 3.1.6.

### 2.3. Stimuli

Two types of on-board movies were used as stimuli: movies containing digit-based road markings (“50” for speed limits) and movies containing road markings based on Japanese characters conveying warning messages to drivers (e.g., “Slow Down”). These movies were selected because digits and characters represent the standard formats for meaningful road markings [9,21]. The character road markings varied in character count (4–9 characters) and semantic content (warning message and a place name). This variation was intentionally retained to examine how fixation metrics change with character count under conditions reflecting real-world road markings, rather than with artificially standardized text. Initially, 10 movies were prepared for each condition; however, owing to unsuitable fixation data in one movie from each condition, nine movies were ultimately analyzed (Table S1).

All movies were filmed during daytime on straight roads in environments that were unfamiliar to the participants. To minimize contextual clues, the on-board movies used as stimuli were carefully selected to exclude as much environmental information, such as curves or intersections, as possible that might allow participants to infer the meaning of the road markings. Additionally, all road-marking movies were recorded from a first-person driver’s perspective using an in-vehicle camera, and any extraneous cues such as navigation voices or subtitles were removed to replicate natural visual conditions. The road markings were designed in accordance with Japanese regulations [7]: the digits measured 5000 mm in height and 1200 mm in width, whereas each character measured 2400 mm in height and 800 mm in width.

The visual recognition time was defined as the period during which a road marking remained within a visual recognition distance of 70 m [7] divided by the driving speed shown in the on-board movie. For digit road markings, this time directly reflected the driving speed, whereas for character markings, it varied depending on the number of characters.

**Figure 1:**
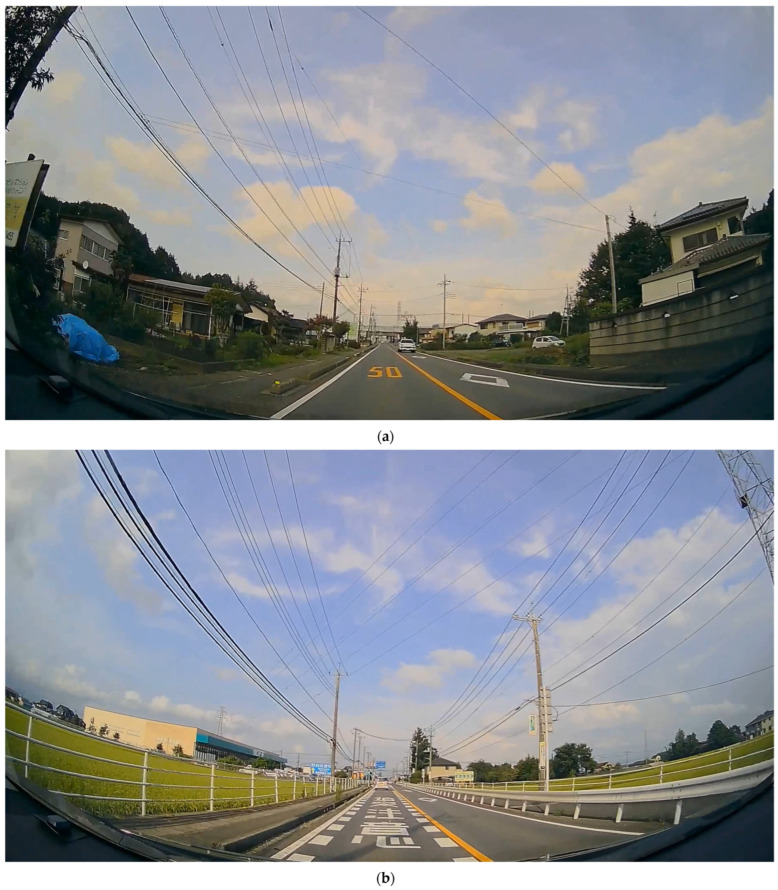
Examples of road markings in the movie. (**a**) Digital road markings; “50” is written in yellow letters on the road surface. (**b**) Character road markings; “追突注意” [Beware of rear-end collision] is written vertically in white letters on the road.

Details of each movie type are provided in Table 1 and Table 2, and example images are shown in Figure 1.

### 2.4. Fixation Behavior Assessment

The fixation behavior was evaluated based on previous research on driving and road marking recognition [22,23,24,25,26,27]. Four indices of fixation were analyzed: (1) the fixation position (x- and y-coordinates in pixels), which represents the spatial location of gaze and its variability [24]; (2) the fixation dispersion (standard deviation of x- and y-coordinates), which indicates the spread or extent of the fixated area [27]; (3) the fixation duration (in milliseconds), which represents the time spent fixating on road markings—longer durations suggest greater attentional allocation [8]; and (4) the number of fixations, which indicates the frequency of attention shifts [22]. The “mean fixation location” was calculated purely by averaging the fixation coordinates, without weighting by fixation duration. Fixations were detected using Tobii Studio’s I-VT filter [28], with the minimum fixation threshold set to 60 ms, based on binocular eye-tracking data.

### 2.5. Cognitive Function Assessment

The following assessments were used to evaluate cognitive functions relevant to driving:

- The Trail Making Test—Japanese Edition (TMT-J) A and B was used to assess attention and processing speed [29]. In Part A, participants are required to connect numbers from 1 to 25 in ascending order as quickly as possible by drawing lines between them. Part B involves both numbers (1 to 13) and hiragana characters (あ to し, corresponding to the Japanese phonetic symbols *a* to *shi*). Participants must alternate between numbers and characters in sequence—e.g., 1–あ (*a*)–2–い (*i*)–3–う (*u*)—and connect them accordingly.
- The Wechsler Memory Scale—Revised (WMS-R) was used to evaluate verbal and visual memory [30]. In this study, two WMS-R subtests were administered: Logical Memory, assessing verbal memory through recall of short stories, and Figure Memory, assessing visual memory by reproducing figures shown for 10 s.
- The Zoo Map Test, from the Behavioral Assessment of the Dysexecutive Syndrome (BADS) was used for planning [31]. In this task, participants are required to plan a route while following specific rules. Previous studies have shown that performance on the Zoo Map Test is associated with driving skills [32,33].
- The useful field of view (UFOV) was assessed using the “Double Decision” task from BrainHQ^®^ (San Francisco, CA, USA) [34,35,36], which measures divided attention and processing speed. In this task, one of two cars is briefly presented in the central visual field, while a Route 66 sign simultaneously appears in one of eight peripheral quadrants. To advance to the next trial, participants must correctly identify both the centrally displayed car and the location of the peripheral sign, thereby engaging both focused and divided attention.

These cognitive function assessment measures have been used in previous studies involving driving simulators and on-road driving [37,38].

The cognitive function results are shown in Table 3. The TMT-J scores were recorded as the completion time (in seconds), whereas the WMS-R and BADS were scored using standardized procedures. Performance on the UFOV task was scored based on accuracy, with 1 point awarded for correctly identifying the type of central stimulus and 1 point for correctly identifying the location of the peripheral stimulus.

### 2.6. Data Collection Procedure

**Figure 2:**
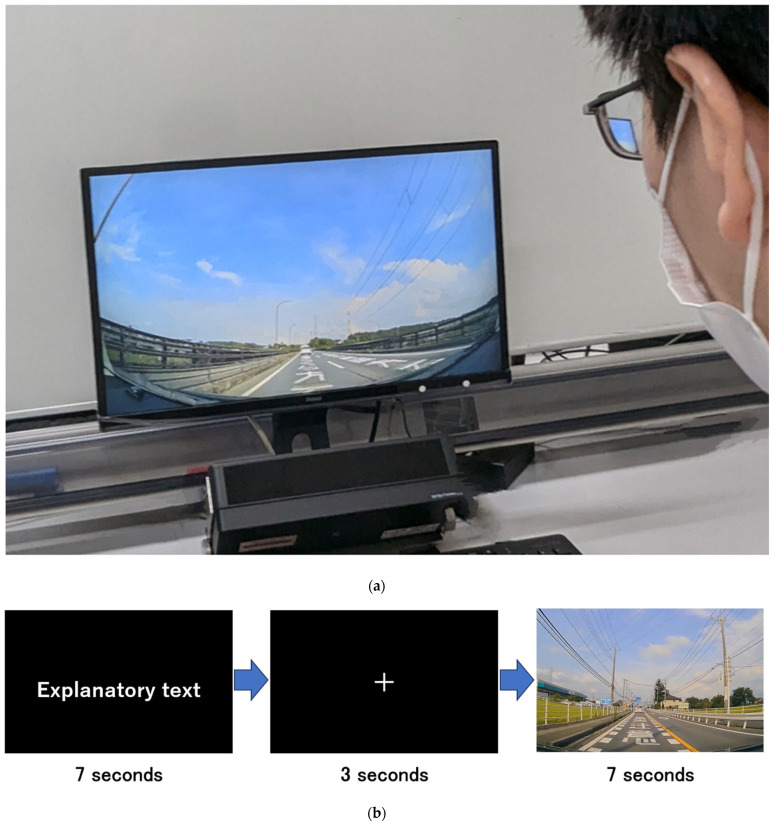
Data collection environment and flow. (**a**) The actual data collection environment, showing a road marking “那須塩原さくら方面” [*Nasushiobara Sakura* Direction]. (**b**) The schematic of the data collection flow, which includes a road marking “追突注意” [Beware of Rear-end Collision]. This flow is repeated nine times in one movie condition.

Before data collection, participants first completed a questionnaire to collect their basic demographics, followed by the cognitive function assessments described in Section 2.5. Participants were seated 57 cm from the monitor, based on a previous study [39] that used on-board movies for a hazard perception task. To better replicate a naturalistic driving environment, no chinrest or any other device was used to restrict head movements. A five-point calibration was conducted prior to data collection. The participants were instructed to watch the road marking videos as if they were driving [5]. Each trial began with a 7-s explanatory text, followed by a 3-s fixation cross and then a 7-s on-board movie. The eye tracker recorded both the fixation duration and number of fixations. This sequence was repeated for each movie, and recalibration was performed before each condition. After each condition, the examiner also confirmed whether participants had recognized all the road markings presented in the movies. The data collection procedure is illustrated in Figure 2.

### 2.7. Statistical Analysis

Analysis 1: Fixation behavior before, during, and after visual recognition of road markings Repeated-measures ANOVAs were conducted on the mean position and dispersions of the fixations (x- and y-coordinates) to examine the fixation behavior before, during, and after the recognition of road markings, as well as to compare digit-based and character-based markings. The analysis followed a 2 (type of road marking: digits vs. characters) × 3 (time: before, during, and after recognition) design, resulting in four separate analyses.

Because the “after” period was less than 1 s across all movies (mean: 0.82 s for digits and 0.63 s for characters), a consistent post-recognition window could not be uniformly applied. To address this issue, the post-recognition window (“after”) was simulated by assuming that participants have maintained fixation at the recognized location for 1 s following recognition. This fixed 1-s window was applied uniformly across all trials to standardize the analysis and ensure comparability between conditions. This approach is considered conservative and physiologically plausible, given that saccade latency typically ranges up to 1 s [40], and that the instructional screen appeared immediately after the movie ended.

The mean fixation position was used as an index of the fixation location, while fixation dispersion, calculated as the standard deviation of fixation positions, indicated the spatial variability (spread) of the fixations rather than statistical variance. When no significant interaction was observed, the main effects for the marking type and time were examined separately. Bonferroni corrections were applied for multiple comparisons, and the Greenhouse–Geisser correction was used when the assumption of sphericity for Mauchly’s test was violated.

Analysis 2: Fixation behavior during visual recognition time.

The fixation data were divided into the first, middle, and final thirds of the visual recognition period to assess changes in fixation behavior during visual recognition and to examine differences between digit and character road markings. A 2 (type: digits vs. characters) × 3 (time: first, middle, final third) repeated-measures ANOVA was conducted on the means and fixation dispersions of the fixation points (x- and y-coordinates), resulting in four separate analyses. Here, fixation dispersion refers to the spatial spread of fixation points and should not be confused with statistical variance. The mean duration of each segment was 327.25 ± 44.13 ms (range: 184.6–441.85 ms) for digits and 440.21 ± 121.89 ms (range: 149.01–584.33 ms) for characters. The mean fixation values were used to indicate the fixation position, whereas fixation dispersion, defined as the standard deviation of fixation positions, was used to indicate the spatial variability (spread) of the fixations rather than the statistical variance. The main effects were analyzed when interactions were not significant.

Analysis 3: Relationship between driving speed, number of letters, and fixation behavior.

Pearson’s correlation coefficients were used to examine the relationships between the fixation duration and number of fixations during visual recognition, and either the driving speed (for digit markings) or number of letters (for character markings), as the driving speed was defined based on the visual recognition time.

Analysis 4: Relationship between fixation behavior, cognitive functions, and basic demographics Pearson’s correlation coefficients were calculated between the fixation duration and number of fixations, and the age, driving history, education, and cognitive function measures (attention and processing speed [TMT-J A and B], verbal and visual memory [WMS-R], planning ability [Zoo Map Test], and UFOV) of the participants to explore the influence of cognitive functions and basic demographics on fixation behavior during recognizing road marking. Spearman’s rank correlation was used for the Zoo Map Test owing to limited score variability. Analyses were conducted separately for the digit and character road markings.

All statistical analyses were performed using EZR version 1.61 [41], with the significance level set to *p* < 0.05.

## 3. Results

All variables satisfied normality assumptions (the Kolmogorov–Smirnov test).

### 3.1. Fixation Behavior Before, During, and After Visual Recognition of Road Markings

No significant interaction was observed between the road marking type and time for either the fixation position or dispersion. A main effect of time was observed for the y-coordinate of the fixation position (*F*(2, 32) = 58.948, *p* < 0.001), with fixation points that were significantly higher during recognition compared with both before and after recognition (both *p* < 0.001), which means that participants looked further up the road (i.e., towards more distant parts of the roadway) during recognition. No main effect of time was found for the x-coordinate.

For the x-coordinate of the fixation dispersion, Mauchly’s test indicated that the assumption of sphericity was violated. Therefore, a Greenhouse–Geisser correction was applied, and a significant main effect of Time was observed (*F*(1.29, 20.57) = 8.886, *p* = 0.005, partial *η*^2^ = 0.36). Multiple comparisons revealed that horizontal dispersion was significantly reduced during recognition compared with both before (*p* = 0.008) and after (*p* < 0.001), suggesting more focused visual attention. No significant main effects of the road marking type or interactions were observed (see Table 4a for fixation position and Table 4b for fixation dispersion).

It should be noted that the assumption of a consistent post-recognition window did not affect the numerical values reported in Table 4a,b.

### 3.2. Fixation Behavior During Visual Recognition Time

No significant interaction was observed between the road marking type and time for the fixation position or dispersion. However, main effects of both the type and time were observed for the y-coordinate of the fixation position. Participants fixated higher on character markings than on digit markings (*F*(1, 16) = 8.009, *p* = 0.012, partial *η*^2^ = 0.33). The fixation height increased over time, with a significant main effect of time after Greenhouse–Geisser correction (*F*(1.32, 21.10) = 7.806, *p* = 0.007, partial *η*^2^ = 0.33). Multiple comparisons showed significantly higher fixations in the middle and final thirds compared with the first third (first vs. middle: *p* < 0.001; first vs. final: *p* = 0.028), indicating that participants looked further up the road (i.e., towards more distant parts of the roadway) as visual recognition time passed.

A main effect of the time was observed for the x-coordinate of the fixation dispersion (*F* = 3.877, *p* = 0.031), with reduced horizontal dispersion in the final third compared with the first third (*p* = 0.034), suggesting increased visual focus towards the end of recognition. No other significant effects were observed (see Table 5a for fixation position and Table 5b for fixation dispersion).

### 3.3. Relationship Between Driving Speed, Number of Letters, and Fixation Behavior

A significant positive correlation was observed between the fixation duration and visual recognition time for digit markings (*r* = 0.719, *p* = 0.029), indicating that longer recognition times were associated with longer fixations. No significant correlation was found for the number of fixations (Table 6a). A significant positive correlation was observed between the number of characters and the fixation duration (*r* = 0.911, *p* < 0.001), suggesting that longer texts required more sustained visual attention. No significant correlation was found for the number of fixations (Table 6b).

### 3.4. Relationship Between Fixation Behavior, Cognitive Functions, and Basic Demographics

No significant correlations were found between the fixation behavior (fixation duration and number of fixations) and cognitive functions or basic demographic variables for either the digit or character markings. Spearman’s rank correlation for the Zoo Map Test also revealed no significant associations (Table 7a for digit road markings and Table 7b for character road markings).

## 4. Discussion

This study analyzed fixation behavior in response to road markings. The results revealed that: (1) there were no significant differences between digit and character markings (type); (2) participants primarily recognized road markings during the latter phase of the visual recognition period; and (3) the fixation behavior was influenced by the driving speed and number of characters.

### 4.1. Fixation Behavior of Road Markings (Digits and Characters) Regardless of the Difference Between the Types

The analysis of the fixation behavior showed that fixations were more horizontally dispersed before and after visual recognition, whereas they were more vertically aligned, with reduced horizontal dispersion, during recognition. This suggests that while drivers temporarily focus their attention on the target when viewing road markings, they tend to distribute their fixations more broadly at other times to monitor their surroundings for safety (e.g., checking for vehicles or pedestrians) [42,43]. In addition, the central placement of road markings within the driver field of vision likely allows for simultaneous peripheral monitoring, thereby supporting situational awareness.

The similarity in eye movement patterns and fixation focus when recognizing digit and character markings may be attributed to the use of high-brightness, high-chroma colors (e.g., white and yellow) [44]. These visual characteristics likely enhanced the visibility and contributed to the lack of significant differences in visual recognition between the two marking types.

### 4.2. Fixation Behavior of Road Markings over Time During Recognition

The analysis of the three visual recognition times showed that the fixation positions moved vertically during the latter phase of recognition and that the horizontal dispersion decreased. That is, we observed a process of gradually focusing the fixation on the markings over time. This is consistent with the “two-stage model” of visual information processing: locating the target in the initial stage, followed by focusing on that target in the second stage [45,46,47]. The mean recognition times for the three segments in this study (digits: 327 ms; letters: 449 ms) align with the processing time described in this model, suggesting a recognition process that starts with identifying the target location and then shifts to focused attention.

Participants also fixated on character markings at a higher position than digit markings throughout the recognition period, likely because the character markings were longer and contained more information. The character markings ranged from four to nine characters and required more detailed visual recognition, whereas the digit markings consisted of only two digits. In addition, character road markings have a clear purpose for their placement, which facilitates directing attention toward them. Because their meaning is explicit and predictable, participants likely understood the message after seeing only the first character.

### 4.3. Effects of Driving Speed and Amount of Information on Fixation Behavior

Furthermore, the results suggest that the driving speed and amount of information contained in the markings influence fixation behavior, particularly the fixation duration. Specifically, the longer the visual recognition time for digit markings, the longer the fixation duration. This suggests that the changes in fixation duration reflect not simply the length of fixation duration, but qualitative changes in attention related to cognitive load and processing complexity. A greater number of characters (i.e., more information) was associated with longer fixation durations. This is consistent with previous studies that showed that faster driving speeds are linked to shorter fixation durations [48]. Furthermore, words with more characters require more complex visual processing, resulting in longer fixation durations [49,50], and the same tendency was observed in this study. These findings indicate that both the driving speed and amount of information in the markings should be considered when designing road markings. In particular, short and simple markings are likely to be more recognizable at higher speeds, whereas slightly more detailed markings may be effective in lower-speed situations.

### 4.4. Effects of Cognitive Functions and Basic Demographics on Road Marking Recognition

The results showed that neither the cognitive functions nor the basic demographics of the drivers were correlated with the recognition of road markings. This suggests that road markings can be recognized by drivers without requiring extensive driving experience or significant cognitive effort. Although we did not use a workload assessment scale such as NASA-TLX [51], the experimental environment using on-board movies included factors that likely reduced cognitive load during recognition. Therefore, the conditions of data collection in this study may have minimized cognitive demands, which could explain the lack of significant correlations with cognitive function measures.

One possible explanation is that all road markings in this study were presented on straight roads, which are known to impose lower cognitive demands [52]. In addition, the font size of the digit and character road markings is large: 5000 mm in height and 1200 mm in width for digits, and 2400 mm in height and 800 mm in width for characters [11]. This means that they are easily recognizable even from a distance. These factors may have contributed to the ease of road marking recognition, regardless of the cognitive functions or basic demographics of the drivers.

Furthermore, all participants were neurologically and psychiatrically healthy individuals with no known history of cognitive or mental disorders, and no abnormalities were observed in their cognitive function assessments. This may have led to a ceiling or floor effect in the cognitive scores, thereby reducing the likelihood of detecting significant associations with road marking recognition.

### 4.5. Limitations

This study has several limitations. First, the stimuli used were on-board movies as opposed to real-world driving situations. As a result, factors that influence fixation behavior during actual driving, such as speedometers, mirrors, and in-vehicle visual fields [53], were not replicated. Therefore, this study should be regarded as a preliminary investigation. Future studies should verify whether the fixation patterns identified here—particularly the two-stage fixation pattern—are reproducible in simulation or real-world driving environments that involve active vehicle control, and should further examine fixation behavior under more realistic driving conditions.

Second, although the sample size was determined a priori using G*Power and data were collected from a comparable number of participants, the statistical power may have been insufficient to detect moderate or small effects, such as Type × Time interactions or correlations with cognitive and demographic variables. While the sample size was likely sufficient to detect large within-subject effects, this limitation implies that some meaningful relationships may have gone undetected. Given that this study is the first exploratory investigation into the relationship between road marking recognition and fixation behavior, the findings nonetheless provide a valuable foundation for future research.

Third, the non-significant correlation between fixation behavior during road marking recognition and the cognitive functions or basic demographics of participants may indicate that the cognitive demands of the task were low, possibly because the markings were presented on straight roads. Yet this possibility could not be tested, as the cognitive load was not quantitatively assessed using tools such as the NASA-TLX [51]. This limitation reduces the interpretability of the findings. Future studies should incorporate quantitative assessments of the cognitive load in road marking recognition and other driving-related tasks.

Finally, although the road markings employed in this study included visual messages such as “Slow Down,” which is intended to encourage deceleration, their specific effects on fixation behavior were not assessed. Previous research has indicated that such markings may induce changes in pupil diameter [54], which suggests that they could influence visual attention through emotional or salience-based mechanisms. In addition, semantic variation among the road markings may have influenced fixation behaviors, which represents a potential confounding factor. Therefore, future studies should explicitly distinguish between different semantic categories, such as deceleration messages and neutral markings, and conduct comparative analyses to more effectively understand their differential impact on fixation behavior.

## 5. Conclusions

The results of this study indicate that drivers exhibit sequential visual behavior when recognizing road markings. During the early phase of visual recognition, they broadly scan both the markings and their surroundings, whereas in the latter phase, they focus more intently on the markings themselves. Marking design should align with the natural progression of visual attention based on this fixation pattern. When multiple pieces of information are included, simple keywords and symbols should be placed at the center or lower part of the markings for initial recognition, while detailed instructions should be positioned at the top, allowing drivers to process them as their fixation shifts and concentration increases.

As fixations tend to move slightly upwards during recognition, particularly critical information in character markings (e.g., “STOP” or “CAUTION: INTERSECTION”) should be placed higher to guide the fixation naturally. Conversely, secondary information (e.g., distance indications or explanatory text) should be positioned lower to align with eye movement patterns.

## Figures and Tables

**Table 1:** Movie features for visual recognition of digit road markings.

	Presence of Cars in Front	Visual Recognition Time (Seconds)
Movie 1	Present	1.36
Movie 2	Absent	1.37
Movie 3	Present	1.34
Movie 4	Absent	1.34
Movie 5	Absent	1.25
Movie 6	Absent	1.5
Movie 7	Absent	1.21
Movie 8	Present	1.21
Movie 9	Present	1.35

**Table 2:** Movie features for visual recognition of character road markings.

	Presence of Cars in Front	The Number of Letters	Written Marking	Visual Recognition Time (Seconds)
Movie 1	Absent	5	Intersection Warning	1.52
Movie 2	Present	9	*Nasushiobara Sakura* Direction	3.32
Movie 3	Present	4	Beware of Rear-end Collision	1.45
Movie 4	Present	5	Slow Down	1.74
Movie 5	Present	4	Beware of Rear-end Collision	1.81
Movie 6	Present	5	Beware of Pedestrians	1.47
Movie 7	Present	5	Curve Warning	1.76
Movie 8	Absent	4	Beware of Rear-end Collision	1.48
Movie 9	Present	5	Slow Down	1.53

**Table 3:** Details of the cognitive functions of participants.

Assessment Measures	Score (Mean ± Standard Deviation (Range))
TMT-J A (seconds)	33.35 ± 11.88 (22.61–69.15)
TMT-J B (seconds)	64.88 ± 48.08 (29.1–260)
WMS-R verbal memory	23.05 ± 5.11 (10–30)
WMS-R visual memory	37.35 ± 4.26 (27–41)
BADS Zoo Map Test	15.5 ± 0.67 (14–16)
UFOV	50 ± 2.95 (43–56)

**Table 4:** (a) Comparison of coordinates of road markings for fixation before, during, and after visual recognition. (b) Comparison of means of fixation dispersions of road markings before, during, and after visual recognition.

**(a)**									
	**Digit Road Markings**			**Character Road Markings**			**Main Effects**		**Interaction**
	**Before 1 s**	**During**	**After**	**Before 1 s**	**During**	**After**	**Type**	**Time**	
**x-coordinate**	900	915	907	919	919	916	*F* = 1.703 *P* = 0.21	*F* = 0.758 *P* = 0.477	*F* = 0.818 *P* = 0.45
**y-coordinate**	889	915	870	894	929	867	*F* = 1.016 *P* = 0.328	***F* = 58.948** ***P* < 0.001**	*F* = 1.397 *P* = 0.262
**(b)**									
	**Digit Road Markings**			**Character Road Markings**			**Main Effects**		**Interaction**
	**Before 1 s**	**During**	**After**	**Before 1 s**	**During**	**After**	**Type**	**Time**	
**x-coordinate**	50.44	27.56	51.14	51.67	26.79	48.38	*F* = 0.014 *P* = 0.908	***F* = 8.886** * P = 0.005 *	*F* = 0.036 *P* = 0.926
**y-coordinate**	41.26	49.90	50.77	53.79	46.15	46.85	*F* = 0.074 *P* = 0.789	*F* = 0.026 *P* = 0.974	*F* = 1.38 *P* = 0.266

Note: Bolded sections indicate significant differences. “*” indicates values after Greenhouse–Geisser correction. The main effect of “Type” is the value when comparing types of road markings, that of “Time” is the value when comparing time axes, and “Interaction” means the interaction between “Type” and “Time”.

**Table 5:** (a) Comparison of coordinates of road markings in fixation during visual recognition time. Each segment (First third, Middle third, and Final third) corresponds to a mean duration of approximately 327.25 ± 44.13 ms (range: 184.6–441.85 ms) for digit road markings and 440.21 ± 121.89 ms (range: 149.01–584.33 ms) for character road markings. (b) Comparison of means of standard deviations of fixation points on road markings during the visual recognition time. Each segment (First third, Middle third, and Final third) corresponds to a mean duration of approximately 327.25 ± 44.13 ms (range: 184.6–441.85 ms) for digit road markings and 440.21 ± 121.89 ms (range: 149.01–584.33 ms) for character road markings.

**(a)**									
	**Digit Road Markings**			**Character Road Markings**			**Main Effects**		**Interaction**
	**First Third**	**Middle Third**	**Final Third**	**First Third**	**Middle Third**	**Final Third**	**Type**	**Time**	
**x-coordinate**	912	917	917	917	918	921	*F* = 0.349 *P* = 0.563	*F* = 2.694 *P* = 0.083	*F* = 0.409 *P* = 0.668
**y-coordinate**	906	920	919	923	930	934	***F* = 8.009** ***P* = 0.012**	***F* = 7.806** * P = 0.007 *	*F* = 0.616 *P* = 0.485
**(b)**									
	**Digit Road Markings**			**Character Road Markings**			**Main Effects**		**Interaction**
	**First Third**	**Middle Third**	**Final Third**	**First Third**	**Middle Third**	**Final Third**	**Type**	**Time**	
**x-coordinate**	39.63	25.71	17.75	32.06	24.95	23.35	*F* = 0.018 *P* = 0.895	***F* = 3.877** ***P* = 0.031**	*F* = 0.683 *P* = 0.512
**y-coordinate**	47.33	41.69	60.69	46.87	45.87	45.71	*F* = 0.277 *P* = 0.606	*F* = 0.574 *P* = 0.445	*F* = 0.574 *P* = 0.423

Note: Bolded sections indicate significant differences. “*” indicates values after Greenhouse–Geisser correction. The main effect of “Type” is the value when comparing types of road markings, that of “Time” is the value when comparing time axes, and “Interaction” means the interaction between “Type” and “Time”.

**Table 6:** (a) Correlations between fixation and visual recognition time in recognizing digit road markings. (b) Correlations between fixation and number of stimuli in recognizing character road markings.

**(a)**	
	Digit road markings
**Correlation between fixation duration and visual recognition time**	***r* = 0.719** ***P* = 0.029**
**Correlation between number of fixations and visual recognition time**	*r* = −0.25 *P* = 0.517
**(b)**	
	Character road markings
**Correlation between fixation duration and number of stimuli**	***r* = 0.911** ***P* < 0.001**
**Correlation between number of fixations and number of stimuli**	*r* = 0.059 *P* = 0.881

**Table 7:** (a) Correlation between basic demographics, cognitive functions necessary for driving, and fixation metrics of digit road markings. (b) Correlation between basic demographics, cognitive functions necessary for driving, and fixation metrics of character road markings.

**(a)**				
	**Fixation Duration**		**Number of Fixations**	
	***r***	***p***	***r***	***p***
**Age**	0.122	0.608	0.005	0.984
**Driving history**	0.106	0.657	0.016	0.948
**Education**	−0.239	0.310	0.361	0.118
**TMT-J A**	0.055	0.818	−0.053	0.825
**TMT-J B**	0.119	0.617	−0.086	0.719
**WMS-R verbal memory**	−0.309	0.185	0.098	0.681
**WMS-R visual memory**	−0.314	0.178	0.254	0.280
**Zoo Map Test**	−0.383	0.096	0.307	0.188
**UFOV score**	0.027	0.911	−0.255	0.278
**(b)**				
	**Fixation Duration**		**Number of Fixations**	
	***r***	***p***	***r***	***p***
**Age**	−0.292	0.212	0.231	0.326
**Driving history**	−0.302	0.195	0.246	0.296
**Education**	−0.241	0.306	0.301	0.198
**TMT-J A**	0.183	0.439	−0.127	0.592
**TMT-J B**	0.290	0.215	−0.201	0.397
**WMS-R verbal memory**	−0.094	0.693	−0.035	0.883
**WMS-R visual memory**	−0.165	0.488	0.053	0.824
**Zoo Map Test**	−0.180	0.448	0.203	0.391
**UFOV score**	−0.071	0.766	0.050	0.834

## Data Availability

The datasets generated and analyzed during this study are not publicly available due to the inclusion of personally identifiable information. However, they may be made available from the corresponding author, Hiroki Okada (e-mail: h-okada@pop.med.hokudai.ac.jp) upon reasonable request and subject to ethical approval. Although the raw data contain personally identifiable information and cannot be shared publicly, anonymized and processed eye-tracking data can be made available to support reproducibility, subject to ethical approval.

## Supplementary Materials

The following supporting information can be downloaded at: https://www.mdpi.com/article/10.3390/jemr18050059/s1, Table S1. Details of the texts written on road markings in each movie.
