# Supplementary material for: Sequential Fixation Behavior in Road Marking Recognition: Implications for Design
Source: J Eye Mov Res. 2025 Oct 21;18(5):59. doi: 10.3390/jemr18050059 (PMC12565692; doi:10.3390/jemr18050059)
Supplement: Supplementary file 1 [file jemr-18-00059-s001.zip › jemr-3792305-supplementary.pdf]

Supplementary Table S1. Details of the texts written on road markings in each movie

Movie	Texts (in Japanese)
Movie 1	交差点注意
Movie 2	那須塩原さくら方面
Movie 3	追突注意
Movie 4	速度落とせ
Movie 5	追突注意
Movie 6	歩行者注意
Movie 7	カーブ注意
Movie 8	追突注意
Movie 9	速度落とせ
